# Supplementary material for: Stable Transcriptional Repression and Parasitism of HIV-1
Source: Mol Ther Nucleic Acids. 2018 May 1;12:12–8. doi: 10.1016/j.omtn.2018.04.011 (PMC6019856; doi:10.1016/j.omtn.2018.04.011)
Supplement: Document S1. Figures S1–S3 and Table S1 [file mmc1.pdf]

OMTN, Volume *12*

Supplemental Information

Stable Transcriptional Repression and Parasitism of HIV-1

Surya Shrivastava, Paige Charlins, Amanda Ackley, Heather Embree, Boro Dropulic, Ramesh Akkina, Marc S. Weinberg, and Kevin V. Morris

Supplemental materials and methods for “Stable transcriptional repression and parasitism of HIV”

Supplemental figures:

Figure S1

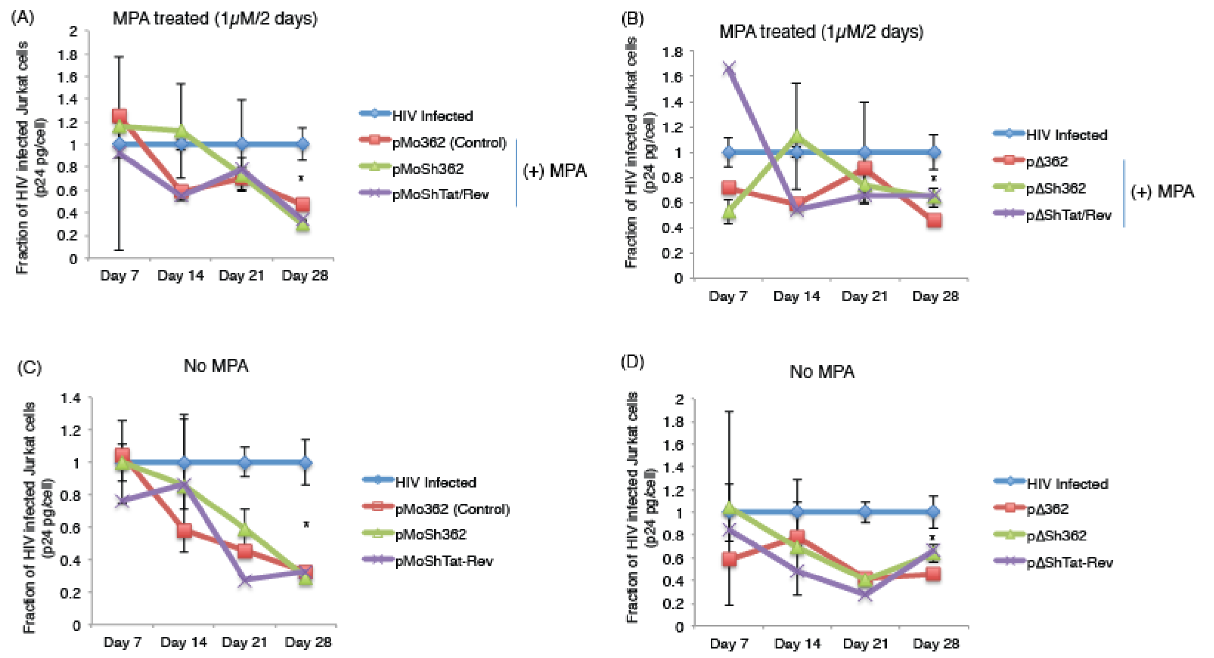

Figure S1 *CR-Vector directed suppression of HIV in Jurkat cells*. Jurkat cells were infected with HIV NL4-3 (MOI-0.01) and 48hrs later transduced with vector (MOI=5.0). The cultures were expanded for 48hrs and then split into 2 groups, one group treated with MPA every 2-3 days (panels A-B) and other group remained untreated (panels C-D). The cultures were followed for 28 days.

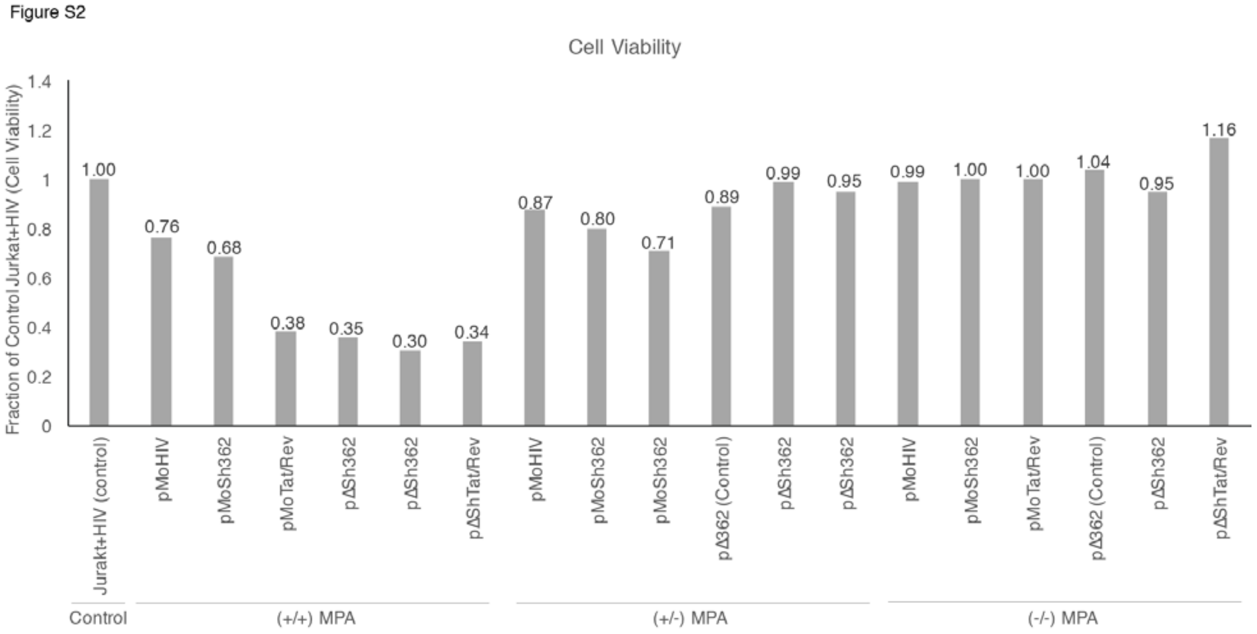

Figure S2 *Cell viability in transduced differential MPA treated and serially passaged cultures.* The cell viability was determined on day 14 for those differentially treated cells shown in Figure 2H. Single measurements are shown from the various cell populations.

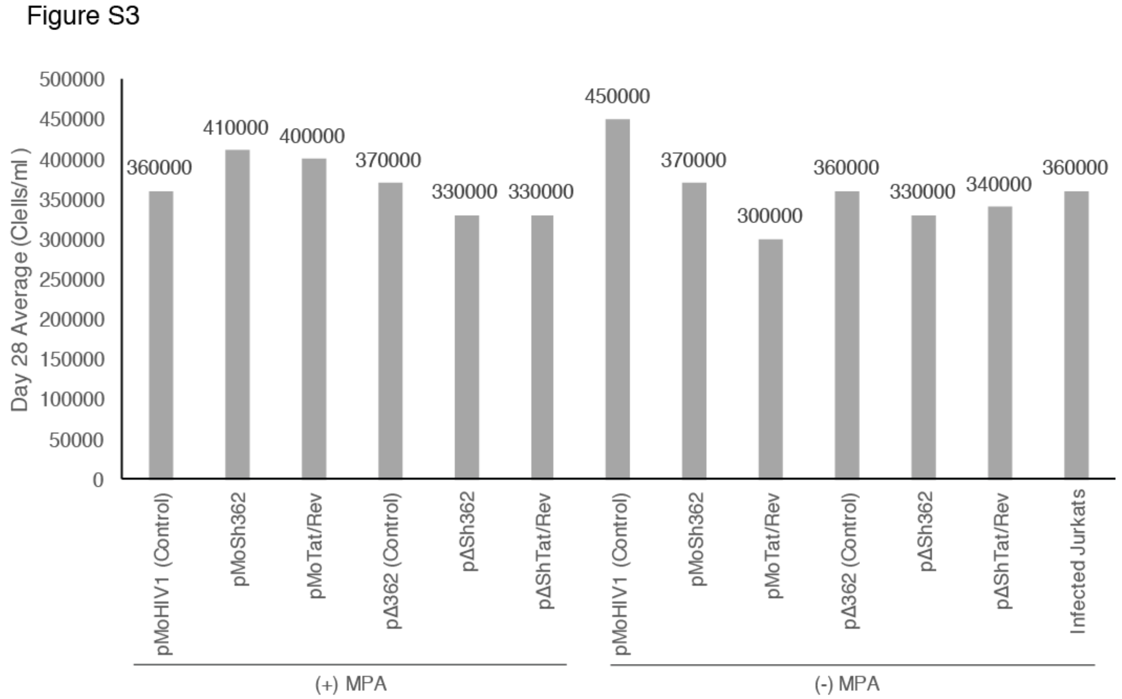

Figure S3 *Cell numbers at day 28 CR-vector treatment.* The average cells were determined 28 days post-CR-vector treatment in HIV infected CR-vector treated Jurkat cells.

Table S1 Oligonucleotide used in the following study.

Name	Sequence (5’-3’)	Function (new name)
GFP F2	GACAACCACTACCTGAGCAC	GFP (vector specific)
GFP R2	CAGGACCATGTGATCGCG	GFP (vector specific)
p128 (HIV F)	AGGGATGGAAAGGATCACCAGCAA	HIV specific
p129 (HIV R)	CCCACCTCAACAGATGTTGTCTCA	HIV specific
p172 (β-actin F)	AGGTCATCACCATTGGCAATGAG	β-actin (house keeping gene)
p173 (β-actin R)	TCTTTGCGGATGTCCACGTCA	β-actin (house keeping gene)
HIV_F1	ACG AGG ATT GTG GAA CTT CTG GGA	HIV specific
HIV_R1	TGG CAT TGA GCA AGC TAA CAG CAC	HIV specific
Surveyor LTR F	GAGAACAACAGCTTGTTACACCCTATGA	LTR-362 specific
Surveyor LTR R	CTGCGTCGAGAGATCTCCTCTGGCT	LTR-362 specific
NFκB LTR_F	TTTCCGCTGGGGACTTTCCAG	LTR-362 site WT sequence specific
Nuc1 LTR_R	ACTCAAGGCAAGCTTTATTGAGGC	LTR nuc1 H3K27me3 site
