# Supplementary material for: Stable Transcriptional Repression and Parasitism of HIV-1
Source: Mol Ther Nucleic Acids. 2018 May 1;12:12–8. doi: 10.1016/j.omtn.2018.04.011 (PMC6019856; doi:10.1016/j.omtn.2018.04.011)
Supplement: Document S2. Article plus Supplemental Information [file mmc2.pdf]

Molecular Therapy
Nucleic Acids
Original Article

Stable Transcriptional Repression and Parasitism of HIV-1

Surya Shrivastava,[1] Paige Charlins,[2] Amanda Ackley,[3] Heather Embree,[4] Boro Dropulic,[4] Ramesh Akkina,[2] Marc S. Weinberg,[3] and Kevin V. Morris[1,3]

[1]Hematological Malignancy and Stem Cell Transplantation Institute and Center for Gene Therapy, City of Hope-Beckman Research Institute, 1500 Duarte Road, Duarte, CA 91007, USA; [2]Department of Microbiology, Immunology and Pathology, Colorado State University, Fort Collins, CO, USA; [3]Molecular and Experimental Medicine, The Scripps Research Institute, La Jolla, CA, USA; [4]Lentigen Technology Inc., Gaithersburg, MD, USA

Gene-based therapies represent a promising treatment for HIV-1 infection, as they offer the potential for sustained viral inhibition and reduced treatment interventions. One approach developed here involves using conditionally replicating vectors (CR-vectors). CR-vectors utilize HIV-expressed proteins to replicate and disseminate along with HIV into the budding viral particles, thereby co-infecting target cellular reservoirs. We generated and characterized several CR-vectors carrying various therapeutic payloads of non-coding RNAs targeted to HIV-1, both transcriptionally and post-transcriptionally. Both virus and vector expression was followed in cell culture systems and T cells in the presence and absence of mycophenolic acid (MPA) selection. We find here that CR-vectors functionally suppress HIV expression in a long-term stable manner and that transcriptional targeting of and epigenetic silencing of HIV can be passaged to newly infected cells by the action of the CR-vector, ultimately establishing a sustained parasitism of HIV. Our findings suggest that CR-vectors with modulatory non-coding RNAs may be a viable approach to achieving long-term sustained suppression of HIV-1, leading ultimately to a functional cure.

INTRODUCTION

For decades, HIV has ravaged human cells. The pathological effect on viral-infected cells stems from the virus itself replicating and interacting with the infected cell. Early models suggest that a 1 to 100 repression of HIV-1 can result in a non-pathogenic state of equilibrium to occur between the virus and the host cells.[1] More recent observations support this notion, whereby an ~1-log reduction in the viral set point can prove therapeutically relevant by doubling the time it takes for the onset of AIDS.[2] One approach outlined here aims to impart significant and sustained reduction of HIV by targeting reservoirs of viral-infected cells using conditionally replicating vectors (CR-vectors).[3, 4]

CR-vectors act as a parasite on HIV and are only functional in those cells that are infected with the replicating virus. Once a CR-vector enters a HIV-infected cell, it can be expressed and packaged, by the actions of Tat and Rev already present within the infected cells, into the budding viral particles and ultimately disseminate along with the virus to newly infected cells.[5] Indeed, CR-vectors functionally add a negative selective pressure on HIV by acting as a defective interfering particle such that the virus is stymied and unable to replicate at high levels.[1] Previous studies have found that CR-vectors containing long antisense transcripts to envelope (ENV) functionally suppress HIV, with up to a 2-log reduction in viral expression in patients, but that compensatory A→G mutations arise quickly in the virus.[6] More recent clinical studies in HIV-infected patients with the same CR-vectors containing long antisense RNAs demonstrated that the vectors are safe, tolerable, and result in reductions in viral set points in 6 out 8 patients.[7] However, the virus was found to elude the selective pressure of the CR-vectors due to A→ G mutations, suggesting that CR-vectors containing smaller therapeutic payloads may avoid A-G mutations and prove more fit at long-term stable suppression of HIV,[7] as has been predicted previously.[1]

Based on the exciting findings that CR-vectors can functionally repress HIV *in vivo*,[7] we developed a new class of CR-vectors that can be selected *in vivo* by the action of the inosine monophosphate to xanthosine monophosphate gene (inosine monophosphate dehydrogenase 2 [IMPDH2]), which is critical for deoxyguanosine triphosphate synthesis.[8] IMPDH2 is preferentially expressed in activated lymphocytes and is 5 times more sensitive to mycophenolic acid (MPA), an inhibitor of IMPDH2, which can be used to selectively expand vector-containing cells.[9] The CR-vectors developed here also contain small non-coding RNA payloads targeted to silence HIV transcriptionally and post-transcriptionally. These non-coding RNA regulatory CR-vectors contain shRNAs targeted to the long terminal repeat (LTR) (LTR-362), a site previously observed to be susceptible to RNA-directed transcriptional gene silencing (TGS),[5,10–14] and an shRNA targeted to the Tat/Rev transcript, a site previously observed to be susceptible to RNAi and post-transcriptional gene silencing (PTGS).[15–17]

Received 2 December 2017; accepted 27 April 2018;
https://doi.org/10.1016/j.omtn.2018.04.011.

Correspondence: Kevin V. Morris, Hematological Malignancy and Stem Cell Transplantation Institute and Center for Gene Therapy, City of Hope-Beckman Research Institute, 1500 Duarte Road, Duarte, CA 91007, USA.
E-mail: kmorris@coh.org

 © 2018 The Author(s).
This is an open access article under the CC BY license (http://creativecommons.org/licenses/by/4.0/).

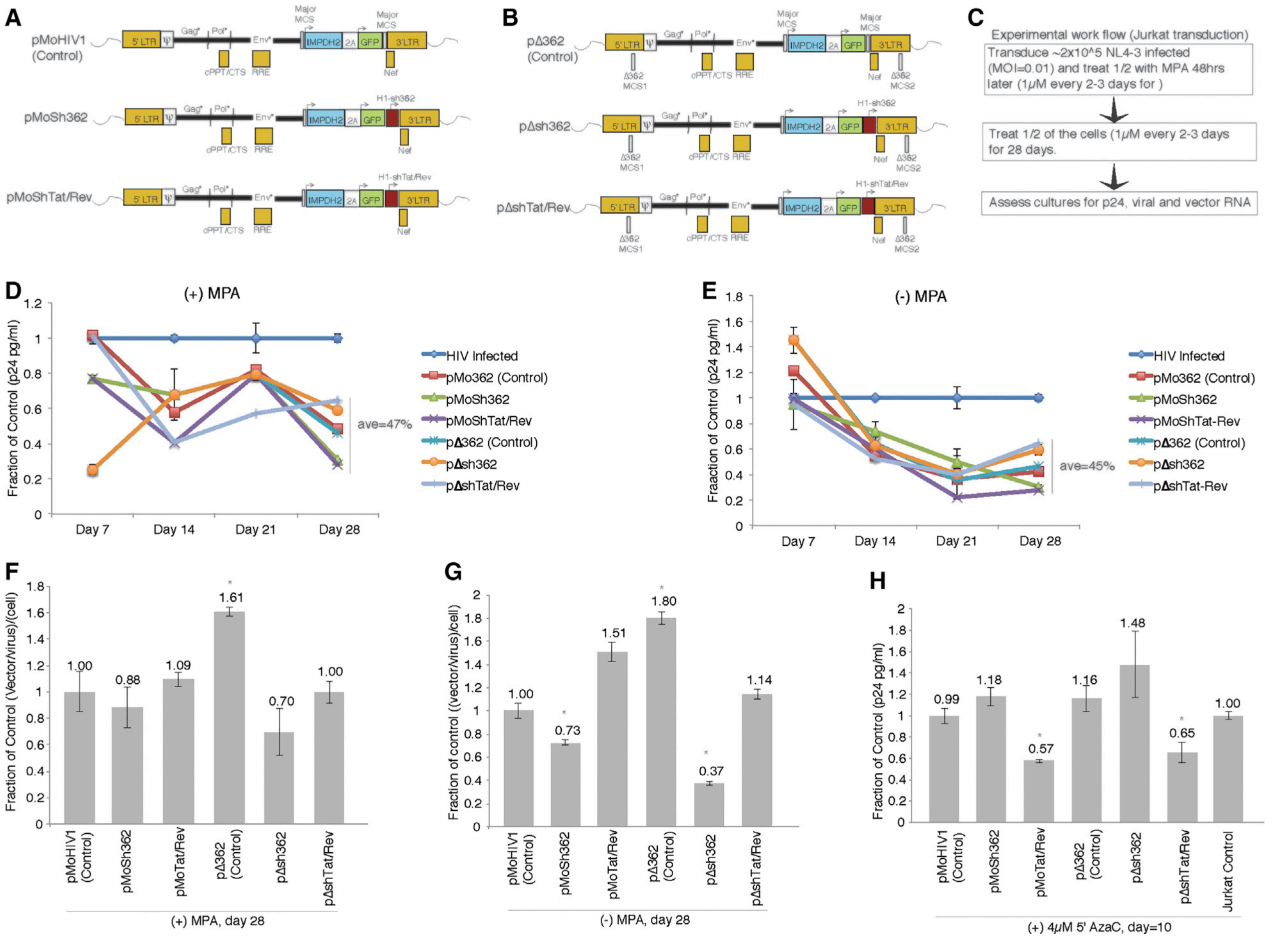

Figure 1. Vector Suppression and Mobilization in HIV-Infected Jurkat Cells

(A and B) Various vectors were generated and tested containing (A) the sh362 target site in the vector LTR (pMoHIV vectors) or with (B) a deletion of the sh362 target site in the LTR (pΔ362 vectors). The vectors also express either shRNA targeted to the LTR-362 locus, sh362, or to the HIV Tat-Rev transcript (shTat/Rev). (C) A workflow is presented depicting how HIV-infected Jurkat cells were treated and assessed for HIV-1 expression and vector mobilization. Jurkat cells were infected with HIV-1 NL4-3 MOI = 0.01, 48 hr later transduced with the vectors (MOI = 5.0), and then 48 hr later treated ±MPA (1 μM every 2 or 3 days) and followed for 28 days for p24 and qRT-PCR for vector and viral content. (D and E) The effects of vector treatment on HIV-1 p24 pg/mL in the presence (D) and absence (E) of MPA. SEMs are shown for the sh362-expressing vectors and control HIV-1-infected Jurkat cells not treated with vector or MPA. (F and G) The relative vector to virus ratio per cell found in the supernatants at day 28 in (F) MPA-treated and (G) untreated cells. (H) The observed repression of HIV-1 by sh362 is blocked by 5′AzaC treatment where shTat/Rev is not affected, suggesting sh362 functions in a transcriptional manner. For (D)–(H), the averages of triplicate-treated cultures are shown with the SDs. *$p < 0.05$ based on a 2-sided paired t test of HIV-infected versus vector-treated cultures.

We find here that CR-vectors alone or with shRNA payloads can, in the presence or absence of MPA, produce stable long-term suppression of HIV-1. We also find here that small-hairpin-RNA-directed TGS of HIV-1 represses HIV-1 long-term, can be co-packaged and passaged from cell to cell with HIV-1, and can maintain epigenetic silencing of HIV-1 following passage. These data presented here suggest that CR-vectors with modulatory non-coding RNAs are a viable approach to achieving long-term sustained suppression of HIV.

RESULTS

One attribute that makes CR-vectors attractive for therapeutic intervention of HIV is that they are parasitic to the virus, as they act as defective interfering particles and co-package with viral transcripts into budding virions.[18] The CR-vectors developed here contained shRNAs targeted to silence HIV-1 either transcriptionally[14,19] or post-transcriptionally[16,17,20] and an IMPDH2 gene that permits inducible MPA selection.[14] The CR-vectors generated and tested here are based on HIV-1 and contain shRNAs targeted to the LTR-362 site in the LTR (sh362)[5,12–14] and the Tat/Rev transcript (shTat/Rev).[21] Two variants of CR-vectors were generated containing an intact LTR and therefore the sh362 target site within the vector (pMoHIV series of vectors; Figure 1A) and those containing a deletion in the LTR-sh362 target site (pΔ362 series of vectors; Figure 1B). The LTR-362 shRNA target site is a locus in the LTR of HIV-1 containing a unique nuclear factor κB (NF-κB) doublet, not found elsewhere in the human genome, which has been observed

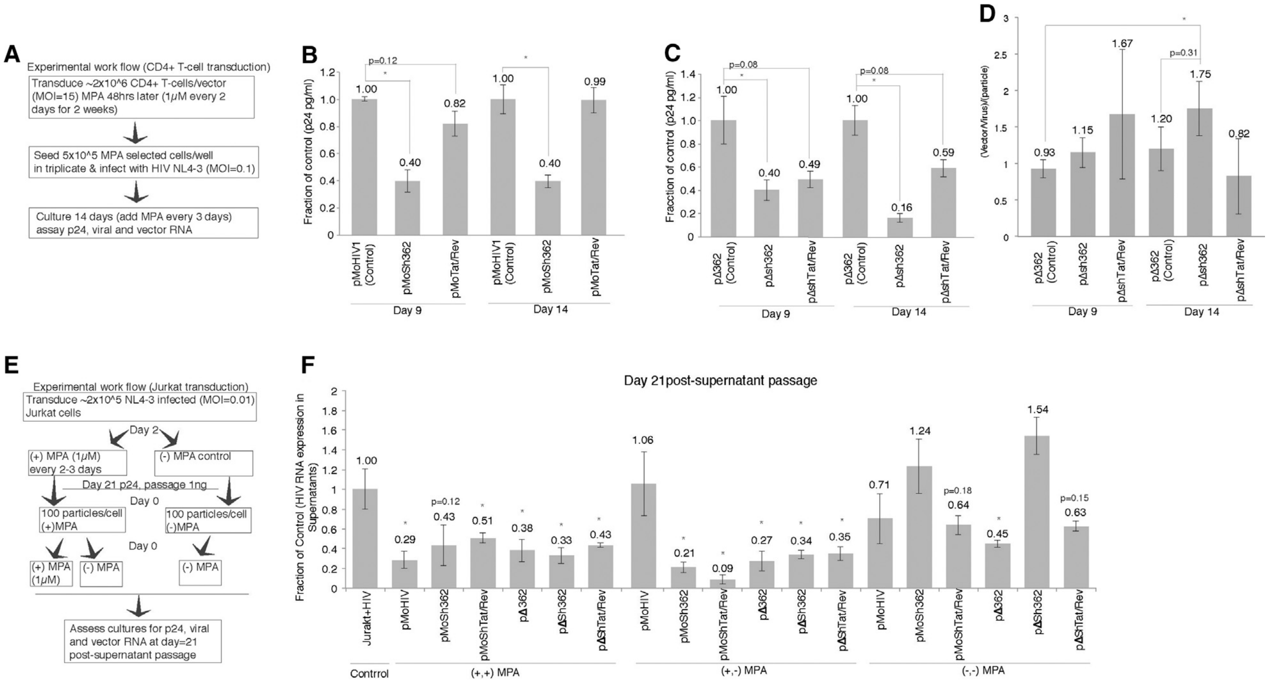

Figure 2. CR-Vector Suppression and Mobilization in HIV-Infected Human Cells

(A) A workflow (for B–D) is presented depicting the CR-vector treatment of CD4+ T cells infected with HIV-1 NL4-3 (MOI = 0.01). (B and C) Both the (B) pMoHIV and (C) pΔ362 small-RNA-expressing CR-vectors repress virus in CD4+ T cells relative to controls. For (B) and (C), the averages of CR-vector-transduced CD4+ T cells infected in triplicate are shown with the SEM and p values from a two-sided paired t test. (D) The ratio of vector to virus per supernatant particle shed into the supernatants at days 9 and 14 in treated CD4+ T cells is shown. The ratio of vector to virus was determined by qRT-PCR and standardized to the number of particles found at days 9 and 14 based on p24 ELISA. The averages of triplicate-treated cultures are shown for each day with the SEMs and p values from a two-sided paired t test. (E) The workflow for determining the effects of MPA on CR-vector selection in HIV-infected Jurkat cells. (F) Cultures supernatants were assessed by qRT-PCR for viral RNA 21 days' post-supernatant passage. The averages of triplicate-treated cultures are shown with the SDs and p values from a 2-sided paired t test. For (B)–(D) and (H), *p < 0.05.

to be highly susceptible to both RNA-directed TGS[10–14,19] and CRISPR-targeted activation of latent HIV.[22–24] Lentivirus-containing CR-vectors expressing anti-HIV shRNAs were generated. These lentiviruses were then transduced into HIV-1 NL4-3-infected Jurkat cells followed by differential treatment with MPA (Figure 1C). Infection in these cultures was followed by p24 until day 28 and assessed for relative CR-vector to virus ratios shed into the cell culture supernatants. Notably, all the CR-vectors tested demonstrated significant long-term suppression of HIV relative to an ongoing viral infection in both the presence (Figure 1D) and absence (Figure 1E) of MPA. The pMoHIV vectors, containing the sh362 target site/NF-κB doublet in the 5′ LTR, appeared to provide enhanced suppression of HIV relative to the pΔ362 vectors as determined by p24 ELISA (Figures 1D, 1E, and S1). Interestingly, all the assessed CR-vectors repressed HIV-1 at similar levels at day 28, and whereas MPA selection didn't appear to enhance the overall suppressive characteristics of these vectors, it did appear to have an effect on cell viability (Figure S2). Notably, the pMoHIV CR-vectors, containing the sh362 target site in the LTR, appeared to exhibit an increased vector packaging potential relative to virus compared to pΔ362 CR-vectors, lacking the sh362 target site in the LTR (Figures 1F, 1G, and S3), presumably because the 362 site is an NF-κB doublet involved in viral transcription[19] and therefore may facilitate packaging of the CR-vectors containing the NF-κB doublet. These observations suggest that the increased suppression of HIV from these vectors and/or the retention of the 362 site in the pMoHIV CR-vectors facilitates greater vector expression and packaging into the virion (Figures 1F and 1G). Indeed, increased packaging of vectors has been observed to limit viral replication and infectivity.[18] Interestingly, whereas MPA demonstrated little effect on suppression of HIV-1 (Figures 1D and 1E), it did affect the ratio of vector packaging such that pΔsh362 CR-vectors could be bolstered in their overall packaging and mobilization potential (Figures 1F and 1G). The addition of the small RNAs, LTR-362, or Tat/Rev-targeting shRNAs may have added a modest enhancement of suppression, specifically with the pMoHIV vectors (Figures 1D and 1E). This suppression with the sh362-expressing CR-vectors appeared to be transcriptional in nature, as the observed repression was lost following 5′Aza cytidine (5′ Aza-C) treatment (Figure 1H). Collectively, these observations suggest the CR-vectors can be packaged and spread out to 28 days post-transduction in HIV-1-infected cell cultures and exhibit long-term silencing capabilities.

Previous studies at clinical level have shown CR-vector-mediated repression of HIV from infected patient CD4+ T cells.[7] To determine the functionality of those CR-vectors developed here, human CD4+

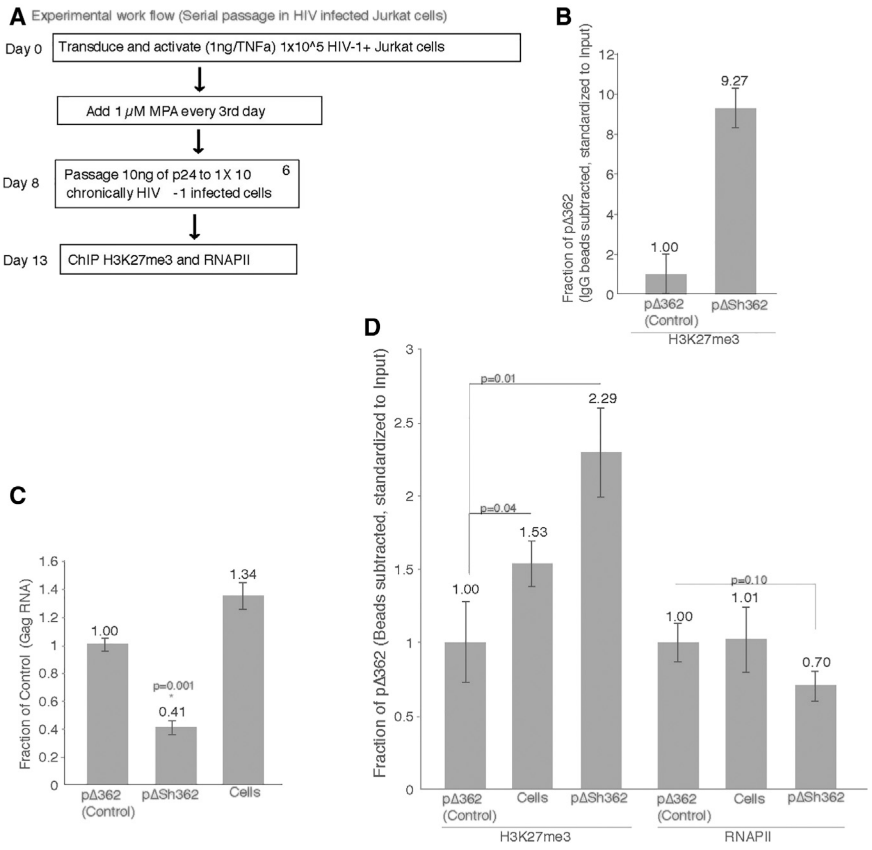

Figure 3. Serial Passage of CR-Vector-Directed Epigenetic Gene Silencing

(A) The workflow for serial passage of CR-vectors in HIV-1-infected Jurkat cells. HIV-1 NL4-3 chronically infected Jurkat cells were transduced (MOI = 5.0) with pΔ362 (control) parent vector or pΔSh362 and then stimulated with 10 ng/mL of TNF-α to activate viral transcription. On every third day, 10 ng/mL TNF-α containing RPMI was added to cells. MPA selection was carried out by adding MPA (1 μM) every third day. (B) ChIP analysis for enrichment of H3K27me3 at the LTR was carried out on the cells at day 10. (C) Viral RNA from culture supernatants was quantitated by qRT-PCR on day 5 after passage of supernatant from CR-vector-transduced cells. (D) ChIP was performed on 1×10^6 chronically infected Jurkat cells (cells), treated with supernatants from 8 days' post-transduction (Figure 4A; ∼10 ng of p24). Supernatant-treated, pΔ362 (control) parent vector and pΔSh362 were contrasted with control-virus-infected cells for H3K27me3 and RNAPII at the HIV-1 LTR. For (B) and (D), the averages of triplicate-treated cultures are shown with the SDs and p values from a paired t test. *$p < 0.001$.

T cells were transduced, MPA selected, and infected with HIV-1 NL4-3 (Figure 2A). Significant repression of HIV-1 was observed after 14 days in culture for both the pMoHIV1 (Figure 2B) and pΔ362 (Figure 2C) CR-vectors. Notably, similar dynamics of CR-vector to virus packaging dynamics were observed in these human CD4+ cells (Figure 2D) as were found in cell culture lines (Figures 1F and 1G). These observations demonstrate that those CR-vectors developed here functionally repress HIV-1 in the context of virus-infected human CD4+ T cells.

To determine to what extent the CR-vectors developed here can not only package into budding virions but also maintain repression of HIV, transduced and HIV-1-infected Jurkat cells were selected with and without MPA and the subsequent viral infection followed over a serial passage. To determine to what extent this level of repression can be serially passaged, a total of 100 particles per cell from day 21 of either ± MPA (Figures 1C–1E) were serially passaged to uninfected Jurkat cells (Figure 2E). Sustained inhibition of HIV-1 was observed at 21 days' post-serial passage (Figure 2F). Notably, MPA treatment proved useful in selecting for all CR-vectors to exhibit similar repressive effects, including the control vectors, which are mobilization competent but lack small RNA inhibitory mechanisms (Figure 2F). Collectively, these data suggest that the CR-vectors developed here are MPA selective and mobilization competent, capable of spreading a ∼50% repression of HIV-1 sustainably in human cells.

The CR-vectors containing the shRNA targeted to LTR-362 site in the viral LTR demonstrated potent mobilization and spread of gene repression. This form of repression has been shown previously to be transcriptional and epigenetic in nature.[5] To determine the extent of mobilization and spread of transcriptional silencing by sh362 vectors across the passage, supernatant from CR-vector-transduced, chronically HIV-1-infected cells were serially passaged on un-transduced but chronically infected HIV-1-infected cells (Figure 3A). A pronounced enrichment of histone 3 lysine 27 tri-methylation (H3K27me3), a hallmark of stable epigenetic silencing,[25,26] was observed on day 10 in these cells (Figure 3B). To determine to what extent this H3K27me3 mark can be serially passaged by the action of the CR-vector, 10 ng of p24 (∼1×10^6 viral particles; ∼10^4 virus particles per picogram of p24) from day 8 was serially passaged to chronically infected Jurkat cells. Cells receiving supernatant from sh362 CR-vector-transduced chronically infected cells demonstrated robust repression of HIV-1 (Figure 3C). A notable enrichment of the H3K27me3 epigenetic mark and significant loss of RNA polymerase II (Pol II) occupancy on HIV-1 LTR promoter was observed at day 5 in these cultures (Figure 3D). These observations suggest not only that CR-vectors can sustainably passage small-hairpin-RNA-targeted epigenetic silencing of HIV-1 but also that the passage of epigenetic information can be transmitted by the action of viral-like particles parasitizing HIV.

DISCUSSION

CR-vectors have been of interest as a treatment strategy for HIV, as they offer the ability to exhibit long-term persistence in a viral-infected population. Evolutionary modeling of CR-vector and viral

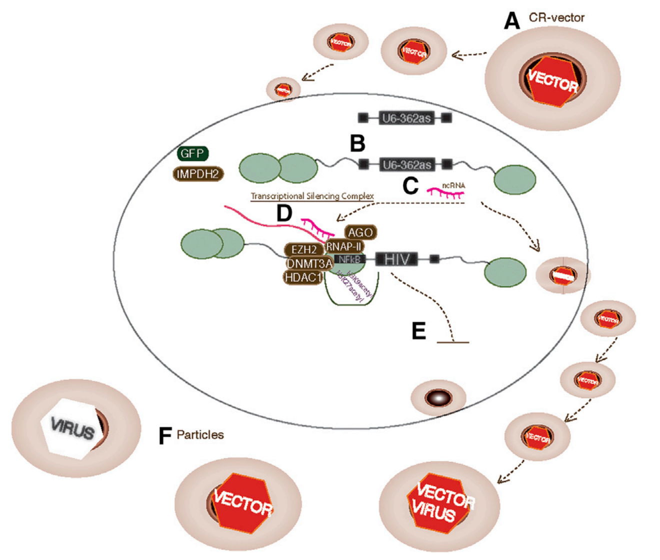

Figure 4. CR-Vector-Mediated Suppression of HIV

(A) CR-vectors are generated and used to transduce infected cells, where they (B) integrate into the genome and (C) express small non-coding RNAs (ncRNA) that can (D) suppress HIV transcriptionally or post-transcriptionally (not shown), resulting in (E) suppression of HIV expression. (F) The CR-vectors can also be expressed and co-packaged into budding virions and spread along with HIV to newly infected cells.

infections suggest that, whereas the CR-vectors will select for resistant variants of HIV, they eventually over time “catch up” and continuously maintain a selective pressure on wild-type HIV.[27] Comparative studies in modeling CR-vectors indicated that an equilibrium is established between the CR-vector and the wild-type replicating HIV.[27,28] This equilibrium results in “red queen” co-evolutionary dynamics,[27] such that the virus must consistently evolves around the selective pressures of the CR-vector, but also that the CR-vector will evolve to be packaged and spread by the actions of wild-type virus. The red queen co-evolutionary dynamics could result in the emergence of the prisoner’s dilemma, a phenomenon that has been observed in nature with the phi-6 bacteriophage.[29] Phi-6 is a highly mutagenic RNA bacteriophage that has been observed to have defective non-replicating phi-6 variants that can sweep through populations of infected cells and impose a significant loss of fitness on the replicating phi-6.[29] Interestingly, defective phi-6 variants follow a selfish pathway that leads to a loss of fitness on the replicating phi-6, which also directly affects the fitness of the defective phi-6. In essence, defective phi-6[29] and presumably defective HIV-1[27,28] evolve a selfish existence, regardless of the overall fitness of the virus population. Ultimately, such selfish behavior leads to an overall loss of fitness.

The observations reported here follow the generation and testing of CR-vectors containing the IMPDH2 gene and shRNAs targeted to regulate HIV-1 transcriptionally and post-transcriptionally (Figure 4). These CR-vectors were shown to be effective in suppressing virus in cell culture and human CD4+ T cells. Further, this suppression can be manipulated by the action of MPA selection and can be passaged along with replicating HIV-1 to new target cells (Figure 4). The CR-vectors reported here are close to the idealized therapeutic CR-vector, as they can be regulated by MPA selection, can passage along with the virus to new infected cells, and continuously suppress viral transcription in an epigenetic and potentially heritable manner. However, CR-vector-mediated repression does not lead to 100% inhibition of viral expression. Expression of CR-vectors during ongoing HIV-1 infection produces mixed population of particles with ∼1/3 CR-vector/CR-vector, ∼1/3 HIV/HIV, and ∼1/3 CR-vector/HIV packaged transcripts (Figure 4F). Such non-homogeneous viral products diminish the production of infective particles that contain the HIV/HIV transcripts. Hence, these observations suggest that, at best calculations, the CR-vectors reported here would exhibit a ∼84% reduction in overall productive infectious particles, which may prove to be enough to sustainably repress HIV in a manner that is refractory to viral mutation and is both persistent and parasitic on the replicating virus.

Overall, we find that IMPDH2-containing CR-vectors, either alone or with small non-coding RNA modes of gene regulation, are effective modulators of HIV-1 expression and that this effect can be retained in a long-term stable manner with MPA selection. The utility of these vectors in a gene therapy for HIV infection has not yet been realized, but evidence from earlier clinical studies[7] suggests that the vectors derived here could prove significant as a method to stably regulating HIV infection in the absence of antiretroviral drugs.

MATERIALS AND METHODS

Generation of pMoHIV and pΔ362 Vectors

Several conditionally replicating vectors were *de novo* generated (Genwiz, San Diego, CA, USA). The vectors were designed to contain several multiple cloning sites with two series of vectors assessed. The pMoHIV1 vectors contained an intact HIV-1 LTR whereas the pΔ362 series of vectors contained a deletion of the sh362 target site to avoid small antisense RNA targeting of the vectors (Figure 2A).

Lentiviral Vector Production, Transductions, and MPA Selection

The various CR-vectors were produced by co-transfection of 10 μg pMDLg/pRRE, 4 μg pRSV-Rev, 5 μg pMD2.G, 5 μg pTATdsRed, and 15 μg of the various CR-vectors into 293T cells using calcium phosphate as previously described.[30] Vectors were titered on 293T cells and analyzed for GFP expression by flow cytometry (FACSCalibur II, Becton Dickinson) to determine infectious particles (IPs)/mL. Jurkat cells were transduced at an MOI of 1 IPs/cell by spinoculation at 1,000 × *g* for 30 min. Cells were placed under 1 μM MPA (Sigma-Aldrich) selection 48 hr post-transduction. Cells were monitored for GFP expression using flow cytometry (The Scripps Flow cytometry and cell sorting core facility, a fee for service core facility) and analysis with the microscope. Stimulated $CD4^+$ HIV-infected (HIV-1 NL43-wild-type [WT] MOI = 0.01) cells were transduced at MOIs of 30 IPs/cell 2 consecutive days. 72 hr post-transduction, cells were placed under 1 μM MPA selection.

RNA or DNA Isolation and Analysis

Viral RNA and total cellular RNA were isolated according to manufacturer's instructions using the QIAamp Viral RNA Mini kit and the RNeasy Mini kit respectively, automated by the Qiacube (QIAGEN, Valencia, CA). Cellular DNA was isolated using the QIAamp DNA mini kit automated by the Qiacube. All RNA samples subject to qRT-PCR were prepared according to the following procedure. Isolated RNA in nuclease-free water was DNase treated using Turbo DNA-free DNase Kit (Ambion, Austin, TX) according to manufacturer's instructions. Following treatment, samples were subject to RT-PCR using Mu-MLV (Life Technologies) according to instructions. All real-time qPCR was carried out using Kapa Sybr Fast universal qPCR mix (Kapa Biosystems, Woburn, MA) and an Eppendorf Mastercycler ep Realplex. Thermal cycling parameters started with 3 min at 95°C, followed by 40 cycles of 95°C for 3 s and 60°C for 30 s. Specificity of the PCR products was verified by melting curve analysis with the following primers used for vector (GFP F2/R2) and virus (HIV_F1/R1; Table S1).

Vector Transfections

HIV-infected (NL43 MOI = 0.01) Jurkat cells were transfected with the Neon electroporation system (Life Technologies, Carlsbad, CA, USA). A total of 1×10^6 cells were transfected with 10 μg of DNA using setting 22 (voltage 1,400, width 10, and pulse 3). Media was changed 24 hr later.

qRT-PCR Analysis of Gene Expression

To determine the relative expression of the respective vectors to HIV mRNAs qRT-PCR was carried out on cellular RNAs or viral RNA isolated from treated HIV-infected (HXB2) culture supernatants. Cells were transfected using the Neon electroporator (Life Technologies, Carlsbad, CA, USA; ~1 μg/10^6 cells) or transduced with lentiviral vectors (described in Turner et al.[14]). The culture mRNAs were collected at various time points post-treatment and DNase treated (Promega Maxwell, Madison, WI, USA). The culture RNAs were then subjected to RT conversion and qPCR using various primer sets (Table S1). The absolute expression of each transcript was determined relative to a standard curve or relative to RPL10[31] or beta actin cellular RNAs (Table S1) when cellular transcripts were assessed. The relative expression of GFP (vector) to HIV (virus) (Table S1) was determined relative to one another and/or an internal RPL10 cellular transcript.

Cell Culture

Jurkat cells were maintained in RPMI 1640, and 293T cells were maintained in DMEM (Corning) supplemented with 10% fetal bovine serum (FBS) (Life Technologies) and 50 U/mL Pen/Strep (Corning). Primary $CD4^+$ T cells were maintained in RMPI 1640 supplemented with 10% heat-inactivated FBS (Life Technologies), 50 U/mL Pen/Strep, and 30 U/mL human recombinant IL-2 (rIL-2). Isolate $CD4^+$ T cells were stimulated with T cell activation media (BioE) immediately after isolation for 24 hr, and then normal interleukin-2 (IL-2)-containing media was added.

Serial Passage of CR-Vectors in Jurkat Cells

Transduced and HIV-1-infected Jurkat (+/−) MPA-treated cells were plated at a density of 1×10^6 cells/mL. The cultures were washed 24 hr post-infection with Dulbecco's PBS (DPBS) and replaced with 1 mL RPMI. The cultures were followed over a predetermined period of time and supernatant samples collected for p24 analysis and supernatant serial passage, and cell pellets for RNA or DNA isolation were collected at intermediate times during the experiment as well as the final day of the predetermined experiment. The saved supernatants were stored at −80°C until analyzed. Cells were split 3:10 every 3 or 4 days.

CR-Vector-Transduced $CD4^+$ T Cells

CD4+ T cells were transduced with the CR-vectors (MOI = 15), and MPA-selected $CD4^+$ cells (1 μM every 2 or 3 days) were plated at a density of 5×10^5 cells/mL in 1 mL of T cells activation media (BioE). Cells were infected with HXB2 at an MOI of 0.001 $TCID_{50}$/cell and washed 24 hr post-infection. Every 3 days, supernatant samples were collected, and total live cells were determined by trypan blue staining using the Countess Automated Cell Counter (Life Technologies). Cells were then split and re-plated at a density of 5×10^5 cells/mL in 2 mL RPMI. Flow-sorted $CD4^+$ cells were plated at a density of 5×10^5 cells/mL in 1 mL RPMI with CD3 and CD28 beads at a ratio of 1 bead:2 cells. Experimental conditions were the same as stated above.

Serial Passage of CR-Vectors in TNF-α-Treated Chronically Infected Jurkat Cells

Chronically HIV-1-infected Jurkat cells were activated to boost HIV-1 production by addition of 10 ng/μL tumor necrosis factor alpha (TNF-α). 1×10^5 activated cells were transduced with 5 MOI of either CR-vector-derived lentivirus. These cells were subjected to 1 μM MPA selection pressure 48 hr post-transduction. After 8 days of MPA treatment, supernatant was collected, and p24 ELISA was done to estimate the virus output. Supernatant volume equivalent to 10 ng of p24 was passed on 1 × 106 chronically infected Jurkat cells for each CR-vector in triplicate. These cells were cultured for 5 days, and then supernatant was utilized for viral RNA estimation by qRT-PCR while cells were subjected to chromatin immunoprecipitation (ChIP) analysis.

p24 Analysis

Samples p24 analysis was performed by the Translational Virology Core at the UCSD Center for AIDS Research (AI36214) as a fee for service.

ChIP Analysis

ChIP was carried out as described in Lister et al.[32] with anti-trimethyl H3K27 (Upstate catalog 07-449) and anti-RNA polymerase II CTD repeat YSPTSPS (Abcam catalog AB817) using HIV LTR primers (Table S1).

SUPPLEMENTAL INFORMATION

Supplemental Information includes three figures and one table and can be found with this article online at https://doi.org/10.1016/j.omtn.2018.04.011.

AUTHOR CONTRIBUTIONS

Each author contributed equally to this body of work.

CONFLICTS OF INTEREST

The authors declare no conflict of interest.

ACKNOWLEDGMENTS

This project was supported by NIAID grants PO1 AI099783-01 and R01 AI111139-01 to K.V.M.

REFERENCES

1. Weinberger, L.S., Schaffer, D.V., and Arkin, A.P. (2003). Theoretical design of a gene therapy to prevent AIDS but not human immunodeficiency virus type 1 infection. J. Virol. *77*, 10028–10036.
2. Gupta, S.B., Jacobson, L.P., Margolick, J.B., Rinaldo, C.R., Phair, J.P., Jamieson, B.D., Mehrotra, D.V., Robertson, M.N., and Straus, W.L. (2007). Estimating the benefit of an HIV-1 vaccine that reduces viral load set point. J. Infect. Dis. *195*, 546–550.
3. Bukovsky, A.A., Song, J.P., and Naldini, L. (1999). Interaction of human immunodeficiency virus-derived vectors with wild-type virus in transduced cells. J. Virol. *73*, 7087–7092.
4. Dropulić, B., Hĕrmánková, M., and Pitha, P.M. (1996). A conditionally replicating HIV-1 vector interferes with wild-type HIV-1 replication and spread. Proc. Natl. Acad. Sci. USA *93*, 11103–11108.
5. Turner, A.M., De La Cruz, J., and Morris, K.V. (2009). Mobilization-competent lentiviral vector-mediated sustained transcriptional modulation of HIV-1 expression. Mol. Ther. *17*, 360–368.
6. Levine, B.L., Humeau, L.M., Boyer, J., MacGregor, R.R., Rebello, T., Lu, X., Binder, G.K., Slepushkin, V., Lemiale, F., Mascola, J.R., et al. (2006). Gene transfer in humans using a conditionally replicating lentiviral vector. Proc. Natl. Acad. Sci. USA *103*, 17372–17377.
7. Tebas, P., Stein, D., Binder-Scholl, G., Mukherjee, R., Brady, T., Rebello, T., Humeau, L., Kalos, M., Papasavvas, E., Montaner, L.J., et al. (2013). Antiviral effects of autologous CD4 T cells genetically modified with a conditionally replicating lentiviral vector expressing long antisense to HIV. Blood *121*, 1524–1533.
8. Allison, A.C., and Eugui, E.M. (2000). Mycophenolate mofetil and its mechanisms of action. Immunopharmacology *47*, 85–118.
9. Yam, P., Jensen, M., Akkina, R., Anderson, J., Villacres, M.C., Wu, J., Zaia, J.A., and Yee, J.K. (2006). Ex vivo selection and expansion of cells based on expression of a mutated inosine monophosphate dehydrogenase 2 after HIV vector transduction: effects on lymphocytes, monocytes, and CD34+ stem cells. Mol. Ther. *14*, 236–244.
10. Suzuki, K., Shijuuku, T., Fukamachi, T., Zaunders, J., Guillemin, G., Cooper, D., and Kelleher, A. (2005). Prolonged transcriptional silencing and CpG methylation induced by siRNAs targeted to the HIV-1 promoter region. J. RNAi Gene Silencing *1*, 66–78.
11. Suzuki, K., Hattori, S., Marks, K., Ahlenstiel, C., Maeda, Y., Ishida, T., Millington, M., Boyd, M., Symonds, G., Cooper, D.A., et al. (2013). Promoter targeting shRNA suppresses HIV-1 infection in vivo through transcriptional gene silencing. Mol. Ther. Nucleic Acids *2*, e137.
12. Suzuki, K., Ishida, T., Yamagishi, M., Ahlenstiel, C., Swaminathan, S., Marks, K., Murray, D., McCartney, E.M., Beard, M.R., Alexander, M., et al. (2011). Transcriptional gene silencing of HIV-1 through promoter targeted RNA is highly specific. RNA Biol. *8*, 1035–1046.
13. Suzuki, K., Juelich, T., Lim, H., Ishida, T., Watanebe, T., Cooper, D.A., Rao, S., and Kelleher, A.D. (2008). Closed chromatin architecture is induced by an RNA duplex targeting the HIV-1 promoter region. J. Biol. Chem. *283*, 23353–23363.
14. Turner, A.M., Ackley, A.M., Matrone, M.A., and Morris, K.V. (2012). Characterization of an HIV-targeted transcriptional gene-silencing RNA in primary cells. Hum. Gene Ther. *23*, 473–483.
15. DiGiusto, D.L., Krishnan, A., Li, L., Li, H., Li, S., Rao, A., Mi, S., Yam, P., Stinson, S., Kalos, M., et al. (2010). RNA-based gene therapy for HIV with lentiviral vector-modified CD34(+) cells in patients undergoing transplantation for AIDS-related lymphoma. Sci. Transl. Med. *2*, 36ra43.
16. Neff, C.P., Zhou, J., Remling, L., Kuruvilla, J., Zhang, J., Li, H., Smith, D.D., Swiderski, P., Rossi, J.J., and Akkina, R. (2011). An aptamer-siRNA chimera suppresses HIV-1 viral loads and protects from helper CD4(+) T cell decline in humanized mice. Sci. Transl. Med. *3*, 66ra6.
17. Zhou, J., Li, H., Li, S., Zaia, J., and Rossi, J.J. (2008). Novel dual inhibitory function aptamer-siRNA delivery system for HIV-1 therapy. Mol. Ther. *16*, 1481–1489.
18. Morris, K.V., and Rossi, J.J. (2004). Anti-HIV-1 gene expressing lentiviral vectors as an adjunctive therapy for HIV-1 infection. Curr. HIV Res. *2*, 185–191.
19. Weinberg, M.S., Villeneuve, L.M., Ehsani, A., Amarzguioui, M., Aagaard, L., Chen, Z.X., Riggs, A.D., Rossi, J.J., and Morris, K.V. (2006). The antisense strand of small interfering RNAs directs histone methylation and transcriptional gene silencing in human cells. RNA *12*, 256–262.
20. Zhou, J., Swiderski, P., Li, H., Zhang, J., Neff, C.P., Akkina, R., and Rossi, J.J. (2009). Selection, characterization and application of new RNA HIV gp 120 aptamers for facile delivery of Dicer substrate siRNAs into HIV infected cells. Nucleic Acids Res. *37*, 3094–3109.
21. Li, M.J., Kim, J., Li, S., Zaia, J., Yee, J.K., Anderson, J., Akkina, R., and Rossi, J.J. (2005). Long-term inhibition of HIV-1 infection in primary hematopoietic cells by lentiviral vector delivery of a triple combination of anti-HIV shRNA, anti-CCR5 ribozyme, and a nucleolar-localizing TAR decoy. Mol Ther. *12*, 900–909.
22. Saayman, S.M., Lazar, D.C., Scott, T.A., Hart, J.R., Takahashi, M., Burnett, J.C., Planelles, V., Morris, K.V., and Weinberg, M.S. (2016). Potent and targeted activation of latent HIV-1 using the CRISPR/dCas9 activator complex. Mol. Ther. *24*, 488–498.
23. Limsirichai, P., Gaj, T., and Schaffer, D.V. (2016). CRISPR-mediated activation of latent HIV-1 expression. Mol Ther. *24*, 499–507.
24. Zhang, Y., Yin, C., Zhang, T., Li, F., Yang, W., Kaminski, R., Fagan, P.R., Putatunda, R., Young, W.B., Khalili, K., and Hu, W. (2015). CRISPR/gRNA-directed synergistic activation mediator (SAM) induces specific, persistent and robust reactivation of the HIV-1 latent reservoirs. Sci. Rep. *5*, 16277.
25. Berger, S.L. (2002). Histone modifications in transcriptional regulation. Curr. Opin. Genet. Dev. *12*, 142–148.
26. Jenuwein, T., and Allis, C.D. (2001). Translating the histone code. Science *293*, 1074–1080.
27. Ke, R., and Lloyd-Smith, J.O. (2012). Evolutionary analysis of human immunodeficiency virus type 1 therapies based on conditionally replicating vectors. PLoS Comput. Biol. *8*, e1002744.
28. Rouzine, I.M., and Weinberger, L.S. (2013). Design requirements for interfering particles to maintain coadaptive stability with HIV-1. J. Virol. *87*, 2081–2093.
29. Turner, P.E., and Chao, L. (1999). Prisoner's dilemma in an RNA virus. Nature *398*, 441–443.
30. Morris, K.V., Gilbert, J., Wong-Staal, F., Gasmi, M., and Looney, D.J. (2004). Transduction of cell lines and primary cells by FIV-packaged HIV vectors. Mol. Ther. *10*, 181–190.
31. Roberts, T.C., Hart, J.R., Kaikkonen, M.U., Weinberg, M.S., Vogt, P.K., and Morris, K.V. (2015). Quantification of nascent transcription by bromouridine immunocapture nuclear run-on RT-qPCR. Nat. Protoc. *10*, 1198–1211.
32. Lister, N., Shevchenko, G., Walshe, J.L., Groen, J., Johnsson, P., Vidarsdóttir, L., Grander, D., Ataide, S.F., and Morris, K.V. (2017). The molecular dynamics of long noncoding RNA control of transcription in PTEN and its pseudogene. Proc. Natl. Acad. Sci. USA *114*, 9942–9947.

OMTN, Volume *12*

Supplemental Information

Stable Transcriptional Repression and Parasitism of HIV-1

Surya Shrivastava, Paige Charlins, Amanda Ackley, Heather Embree, Boro Dropulic, Ramesh Akkina, Marc S. Weinberg, and Kevin V. Morris

Supplemental materials and methods for "Stable transcriptional repression and parasitism of HIV"

Supplemental figures:

Figure S1

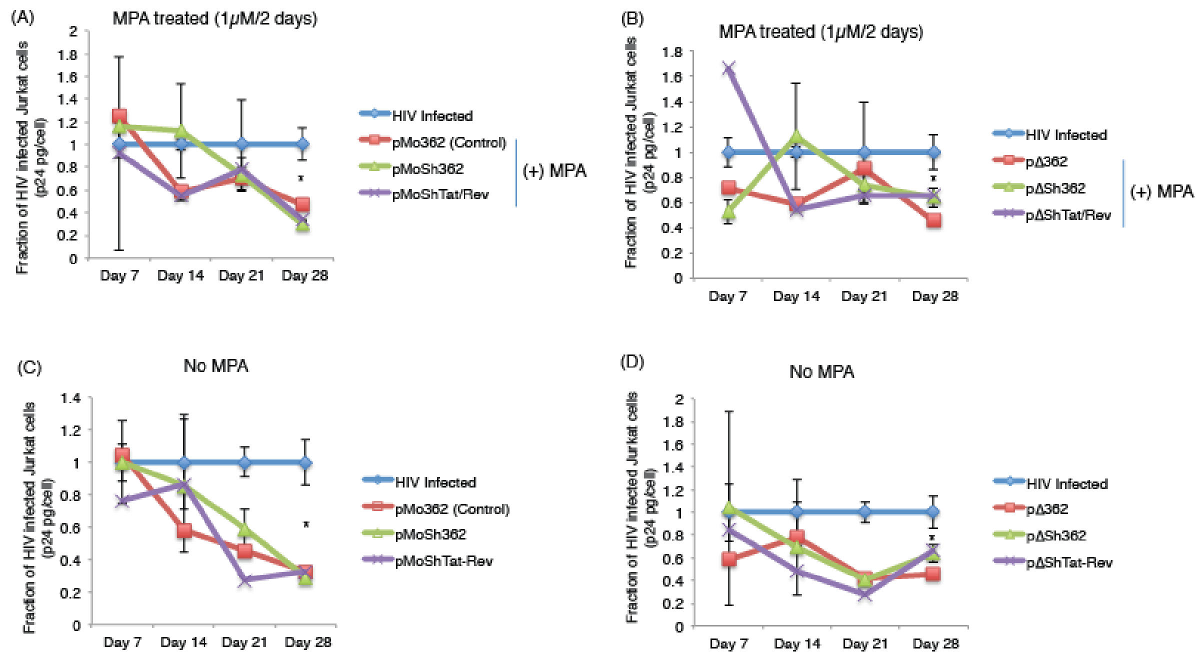

Figure S1 *CR-Vector directed suppression of HIV in Jurkat cells*. Jurkat cells were infected with HIV NL4-3 (MOI-0.01) and 48hrs later transduced with vector (MOI=5.0). The cultures were expanded for 48hrs and then split into 2 groups, one group treated with MPA every 2-3 days (panels A-B) and other group remained untreated (panels C-D). The cultures were followed for 28 days.

Figure S2

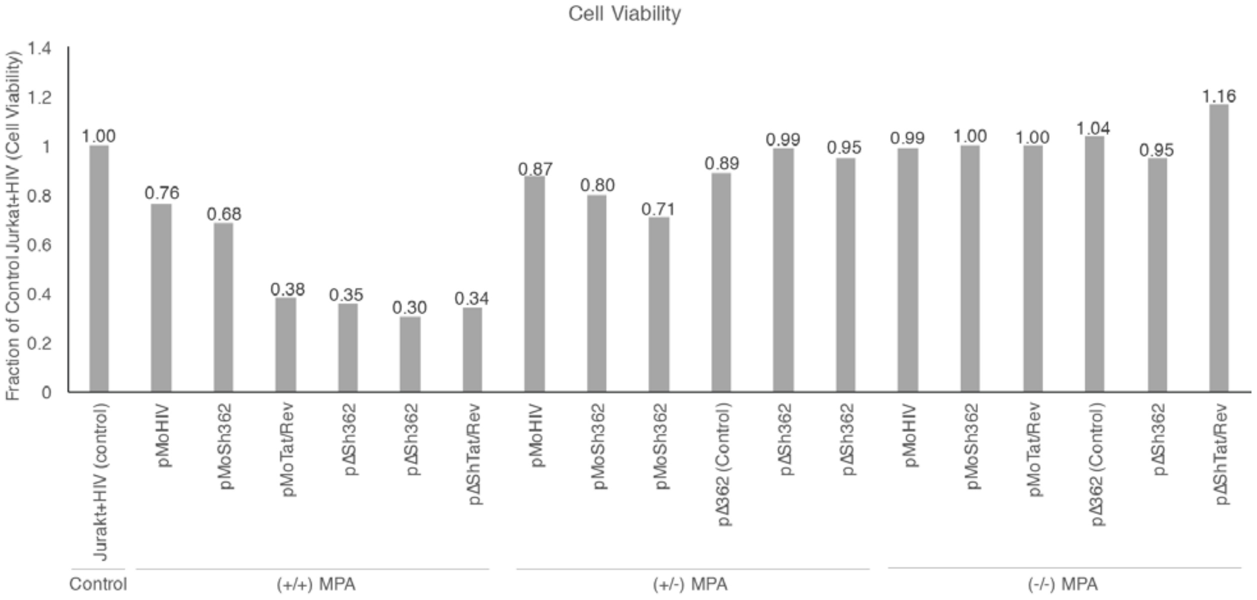

Figure S2 *Cell viability in transduced differential MPA treated and serially passaged cultures.* The cell viability was determined on day 14 for those differentially treated cells shown in Figure 2H. Single measurements are shown from the various cell populations.

Figure S3

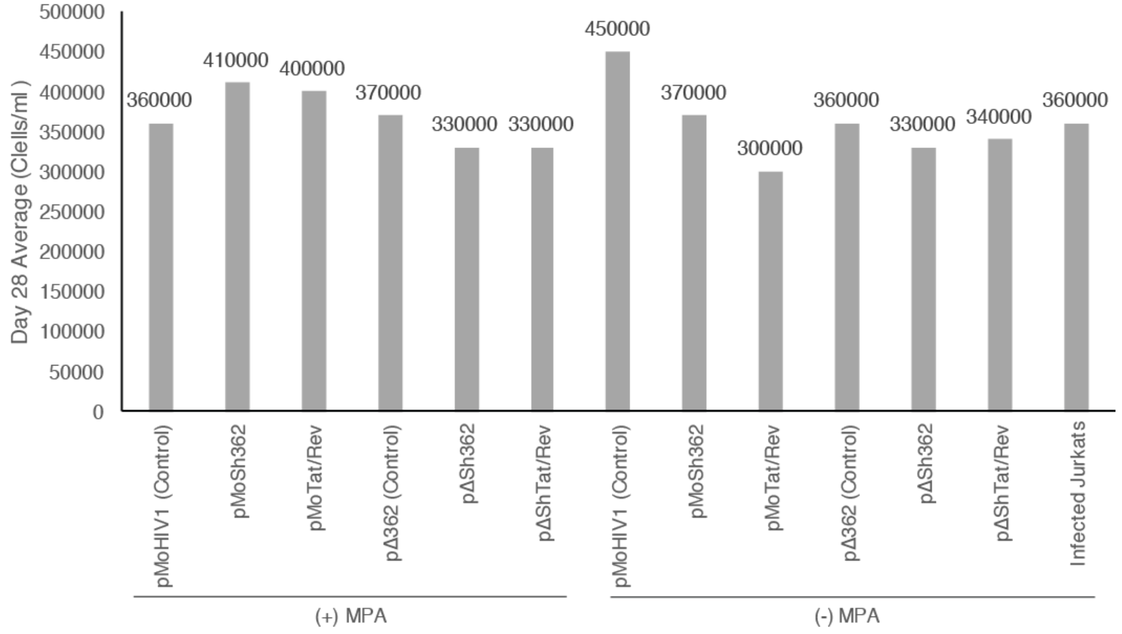

Figure S3 *Cell numbers at day 28 CR-vector treatment.* The average cells were determined 28 days post-CR-vector treatment in HIV infected CR-vector treated Jurkat cells.

Table S1 Oligonucleotide used in the following study.

Name	Sequence (5’-3’)	Function (new name)
GFP F2	GACAACCACTACCTGAGCAC	GFP (vector specific)
GFP R2	CAGGACCATGTGATCGCG	GFP (vector specific)
p128 (HIV F)	AGGGATGGAAAGGATCACCAGCAA	HIV specific
p129 (HIV R)	CCCACCTCAACAGATGTTGTCTCA	HIV specific
p172 (β-actin F)	AGGTCATCACCATTGGCAATGAG	β-actin (house keeping gene)
p173 (β-actin R)	TCTTTGCGGATGTCCACGTCA	β-actin (house keeping gene)
HIV_F1	ACG AGG ATT GTG GAA CTT CTG GGA	HIV specific
HIV_R1	TGG CAT TGA GCA AGC TAA CAG CAC	HIV specific
Surveyor LTR F	GAGAACAACAGCTTGTTACACCCTATGA	LTR-362 specific
Surveyor LTR R	CTGCGTCGAGAGATCTCCTCTGGCT	LTR-362 specific
NFκB LTR_F	TTTCCGCTGGGGACTTTCCAG	LTR-362 site WT sequence specific
Nuc1 LTR_R	ACTCAAGGCAAGCTTTATTGAGGC	LTR nuc1 H3K27me3 site
